# Hematologic manifestations of babesiosis

**DOI:** 10.1186/s12941-017-0179-z

**Published:** 2017-02-15

**Authors:** Tamer Akel, Neville Mobarakai

**Affiliations:** 1 Department of Internal Medicine, Staten Island University Hospital, 475 Seaview Avenue, Staten Island, NY 10305 USA; 2 Department of Infectious Diseases, Staten Island University Hospital, 475 Seaview Avenue, Staten Island, NY 10305 USA

## Abstract

**Background:**

Babesiosis, a zoonotic parasitic infection transmitted by the Ixodes tick, has become an emerging health problem in humans that is attracting attention worldwide. Most cases of human babesiosis are reported in the United States and Europe. The disease is caused by the protozoa of the genus Babesia, which invade human erythrocytes and lyse them causing a febrile hemolytic anemia. The infection is usually asymptomatic or self-limited in the immunocompetent host, or follows a persistent, relapsing, and/or life threatening course with multi-organ failure, mainly in the splenectomized or immunosuppressed patients. Hematologic manifestations of the disease are common. They can range from mild anemia, to severe pancytopenia, splenic rupture, disseminated intravascular coagulopathy (DIC), or even hemophagocytic lymphohistiocytosis (HLH).

**Case presentation:**

A 70 year old immunocompetent female patient living in New York City presented with a persistent fever, night sweats, and fatigue of 5 days duration. Full evaluation showed a febrile hemolytic anemia along with neutropenia and thrombocytopenia. Blood smear revealed intraerythrocytic Babesia, which was confirmed by PCR. Bone marrow biopsy was remarkable for dyserythropoiesis, suggesting possible HLH, supported by other blood workup meeting HLH-2004 trial criteria.

**Conclusion:**

Human babesiosis is an increasing healthcare problem in the United States that is being diagnosed more often nowadays. We presented a case of HLH triggered by Babesia *microti* that was treated successfully. Also, we presented the hematologic manifestations of this disease along with their pathophysiologies.

## Case presentation 

A 70-year-old female patient, who recently immigrated to New York City, United States of America presented to the emergency department in mid-September 2015 for episodic high grade fever associated with confusion, chills, night sweats, fatigue, nausea, headache and palpitations for 5 days. She had moved from South Korea to the United States in June 2015. Prior to presentation, she was prescribed a course of amoxicillin–clavulonic acid for three days for a possible upper respiratory tract infection. Her past medical history is remarkable only for hypertension controlled with hydrochlorothiazide. The patient denied any recent travel history, contact with pets or tick bites and stated that she lives in New York City, and occasionally visits the city’s gardens next to her house. Her social history is only remarkable for smoking half a pack of cigarettes daily; she denied any alcohol or drug use. She had never been hospitalized. In the emergency department she was hypotensive, tachycardic, and febrile with a temperature of 39.3 °C, a pulse of 102 bpm and a blood pressure of 92/58 mmHg. She was awake, alert but not oriented. Physical examination was unremarkable except for disorientation which resolved after becoming normothermic. Her blood pressure responded to 1L of normal saline.

Blood tests showed abnormal cell counts with neutropenia (1.23 × 10^9^/L; ANC <1.5 × 10^9^/L), lymphopenia (0.57 × 10^9^/L; ALC <1 × 10^9^/L), anemia (Hemoglobin of 6.8 g/dL; Hb <12 g/dL), thrombocytopenia (45 × 10^9^/L), and MCV of 85 fL. Serum chemistries were all normal. Liver function tests were only remarkable for a mild elevation in total bilirubin (1.7 mg/dL) with an indirect bilirubin of 1.35 mg/dL. Other blood tests showed elevated C-reactive protein (11.6 mg/dL), LDH of 476 IU/L, ferritin of 1316 ng/mL, reticulocyte production index of 0.7% and an undetectable haptoglobin. A blood smear was ordered on the third day of hospitalization for the evaluation of hemolysis which was only remarkable for schistocytes without any detectable parasites.

She was started on broad spectrum antibiotics for a working diagnosis of sepsis. Blood cultures were sent with no bacterial growth reported few days later. Imaging studies done along with a lumbar puncture were all unremarkable. Febrile hemolytic anemia was our working diagnosis with an infectious etiology being highest on our differential. Testing for HIV, EBV, CMV, Lyme disease, West Nile virus, parvovirus B19, *Anaplasma phagocytophilum* and *Babesia microti* were all sent out, but took at least four days to get reported. Meanwhile, an immunological workup done was also unremarkable. Direct coombs test was negative.

She was actively hemolyzing with persistently daily high grade fevers despite antibiotics. The patient was given steroids for possible coombs negative autoimmune hemolysis without any response. During her 10 day hospital stay she required multiple RBCs’ transfusions to keep the hemoglobin above 7 mg/dL. Bone marrow biopsy was performed on the fourth day of hospitalization which showed dyserythropoeisis. She continued to be pancytopenic; repeat blood smear on the seventh day showed intraerythrocytic Babesia in 4% of the RBCs. Infectious serology testing on blood using IFA sent initially was remarkable for positive IgM antibodies against *B. microti* with a titer of 1:256 along with a positive real-time PCR for the 18S rRNA gene [1]. Atovaquone 750 mg every 12 h orally and Clindamycin 600 mg every 8 h were started and the patient responded well. After 10 days of hospitalization, the patient was discharged, and followed up with an outside physician who reported that she was doing well. Repeated CBC showed resolution of the hemolytic anemia. A follow up peripheral blood smear was clear of hemoparasites.

## Background

Human babesiosis, an emerging zoonosis caused by the hemoparasites of the genus Babesia, the second most common blood-borne parasites of mammals after trypanosomes [2]. Infection of the human host is being diagnosed more often, probably due to increasing number of travelers, immunocompromised individuals, blood transfusions, and better diagnostic methods. The first case of human babesiosis was reported in a Yugoslavian farmer in 1956 [3]. More than 100 Babesia species infect a wide variety of domestic and wild animals, but only few infect humans [4]. The main species of Babesia that are thought to cause the majority of human babesiosis are *Babesia microti*, *Babesia divergens* and *Babesia venatorum* [2, 4]. In the northeastern part of the US, babesiosis is usually caused by the rodent species *B. microti*, which is transmitted by the tick, *Ixodes scapularis*, the same tick vector responsible for the transmission of anaplasmosis and Lyme disease; and thus co-infection with *Anaplasma phagocytophilum* and *Borrelia burgdorferi* should always be considered and tested for [5]. A study of 1000 patients who are seropositive for *B. burgdorferi* found that 10% of them had antibodies to *B. microti* [6]. In Europe, human babesiosis is mainly caused by the cattle species *B*. *divergens* which is also transmitted by the Ixodes tick (*Ixodes ricinus* being the most important) [2]. The incubation period for babesiosis is somewhere between 5 and 30 days [7]. Moreover, the disease has been increasingly acquired over the past decade by blood transfusions; the incubation period in such cases has been reported to be as long as 63 days, and in one case, up to 6 months [7, 8]. The incidence of transfusion-transmitted Babesia has been reported to be about 1.1 cases per million packed RBCs across the United States [9]. More specifically, in endemic areas like Rhode Island, the incidence once approached 1 case per 9000 units of blood transfused [10].

The presentation of the disease is variable, ranging from subclinical, self limited asymptomatic infection to a life threatening one depending on the immune system of the host. Symptoms are usually non-specific and constitutional; initially start as fatigue, generalized weakness, and malaise, followed by abdominal pain, nausea, vomiting, photophobia, and anorexia. Hematuria and jaundice can also be observed depending on the degree of hemolysis. In a review of 139 patients hospitalized with babesiosis in New York, the most common symptoms were: fever (91%), fatigue, malaise, and weakness (91%), shaking chills (77%), and diaphoresis (69%) [11]. Physical examination is usually remarkable for fever, tachycardia or bradycardia, hepatosplenomegaly might be present, and lymphadenopathy is usually absent. The disease is more severe in splenectomized and immunosuppressed patients, and may require multiple blood transfusions or even exchange transfusion. In addition, these patients might relapse and the parasite might persist despite treatment [2].

Babesia parasites can be visualized on blood smears using the Giemsa-Wright stain. They are intraerythrocytic ring forms that resemble plasmodium, the causative agent of malaria. There are a couple of distinguishing features that can hint towards one organism over the other on light microscopy [12]. In Babesia, the parasite can form tetrads or maltese cross, although rare with *B. microti* but pathognomonic of babesiosis. In addition, Babesia does not generate hemozoin (malaria pigment) in the affected RBCs. Nevertheless, hemozoin is also not found during the early trophozoites of plasmodia. Additionally, Babesia has extracellular merozoites [12]. Furthermore, the blood smear might not show the parasites when the degree of RBCs infection is minimal i.e. <0.01%. Light microscopy has excellent sensitivity for Babesia detection, and should only be performed by an experienced microscopist, especially when thick blood smears are done, as the organisms might appear as simple chromatin dots that could be mistaken for a stain precipitate or iron inclusion bodies [13]. In the immunocompetent host, parasitemia can be hard to detect on a peripheral blood smear given that it rarely exceeds 5%, in comparison to the asplenic patient where parasitemia may amount up to 85% [7, 14]. Other diagnostic tests can be used such as real-time quantitative PCR or conventional PCR. The detection limit of PCR is usually 50 parasites per ml, while that of light microscopy is approximately 0.001% parasitemia, which is around 5000 infected erythrocytes per mL [7, 15]. Since babesiosis typically presents with a parasitemia of >0.1%, the assay is exceedingly sensitive for the detection of most clinical specimens [1]. Antibody testing can also be used, since sero-conversion is always required for complete clearance of the parasites [16]. Nearly all infected patients will have detectable antibodies in an acute phase serum sample; this might not be the case in immunocompromised patients, however [13]. Immunofluorescence assays are used to detect titers for *B. microti* in specific. Titers from 1:32 to 1:160 were reported to be both diagnostic and specific, with 88–96% sensitivity, 90–100% specificity [17].

## Discussion

Human babesiosis is increasingly seen more often among the immunocompetent host, especially in the aging population. Age related decline in cellular immunity might help explain the severity of babesiosis in patients older than 50 years of age [4]. This can be reflected by a mouse model which showed age-associated loss of immunity against *B. microti* [18]. The patient we presented was an elderly woman without any other known risk factors. However, she followed a moderate-to-severe course of the disease and became transfusion-dependant on a daily basis for one week.

Clinical manifestations of severe babesiosis can have multiple complications; these include acute respiratory failure, non-cardiogenic edema, congestive heart failure, renal failure, DIC, splenic infarction or even HLH. Complications can occur early or late in the course of the disease. One review of 34 cases of babesiosis who were hospitalized in Long Island confirmed that acute respiratory failure is the most common complication (happened in 7 out of 34 cases), followed by DIC (happened in 6 out of 34 cases) [19].

An extremely rare, yet can be a fatal complication of babesiosis is HLH. HLH is likely an under-diagnosed disease that can result in multi-organ failure and death [20]. It is a condition characterized by excessive inflammation, hypercytokinemia, abnormal immune activation and tissue destruction. This results from a lack of normal down-regulation of activated macrophages and lymphocytes [21]. The dysregulation is due to the inability of natural killer cells and cytotoxic lymphocytes to eliminate the activated macrophages. This dysregulation along with the excessive secretion of cytokines cause tissue damage. HLH is classified into a primary form and a secondary form. The primary form typically manifests in young adults with genetic abnormalities of the cytotoxic function of natural killer cells and T cells. The secondary form of the disease occurs in older people who usually have an underlying condition, such as infection, malignancy or an autoimmune disorder without any identifiable genetic defect. Infectious diseases associated with HLH include epstein-barr virus, cytomegalovirus, human herpes virus-8, herpes simplex virus, varicella-zoster virus, H1N1 influenza virus, measles virus, parechovirus, parvovirus, and HIV. Leishmaniasis has also been reported to precipitate HLH, although more reported in the pediatric population.

The diagnostic criteria have been derived from the HLH-2004 trial [22]. Five out of the 8 criteria listed in Table 1 need to be met to diagnose HLH [22, 23].

**Table 1:** Diagnostic criteria for HLH used in the HLH-2004 trial

Five of the eight criteria listed below should be fulfilled
Fever ≥38.5 °C
Splenomegaly
Cytopenias (affecting at least 2 of 3 lineages in the peripheral blood)
Hemoglobin <9 g/dL (in infants <4 weeks: hemoglobin <10 g/dL)
Platelets <100 × 10^3^/mL
Neutrophils <1 × 10^3^/mL
Hypertriglyceridemia (fasting, >265 mg/dL) and/or hypofibrinogenemia (<150 mg/dL)
Hemophagocytosis in bone marrow, spleen, lymph nodes, or liver^a^
Ferritin >500 ng/mL
Elevated sCD25 (α-chain of sIL-2 receptor)
Low or absent NK-cell activity

^a^Findings in up to two-thirds of initial bone marrow aspirates may be nondiagnostic; an additional bone marrow finding includes dyserythropoiesis, which has been observed in the absence of hemophagocytic histiocytes [24]

It should also be noted that these diagnostic criteria were used in clinical trials, and thus may not identify every single case of HLH. In the literature, there are only 4 cases reported since 1986 with HLH secondary to babesiosis. The first reported case was in a patient with cryptosporidium infection [25]. The second case was in an asplenic renal transplant patient on immunosuppressive therapy [26, 27]. Another case report suggested a possible HLH in a man with amyopathic dermatomyositis and ILD who is on rituximab therapy [28]. The last case report was a possible HLH in an immunocompetent patient who presented with severe pancytopenia and hemophagocytosis on bone marrow biopsy [29]. In our case, the patient had pancytopenia, fever, dyserythropoiesis, hypofibrinogenemia, and a high ferritin level meeting the criteria defined by HLH-2004 trial. Hence, we hypothesize that he had HLH triggered by Babesia. When HLH is triggered by acute infection, and the patient is stable, the appropriate therapy is removal of the stimulus that is activating the immune system. This strategy may allow patients to avoid HLH specific therapy which is potentially toxic and should be reserved for patients who are severely ill. However, in our case, we highlight the role of prednisone given initially, as it is difficult to conclude whether it had a role on the disease itself. Corticosteroids are part of the initial therapy for HLH, since it has a role in controlling the over-activation of the immune system. Conversely, since the precipitating etiology in our case was infectious, it is difficult to tell whether steroids had a role in exacerbating the disease, or whether it was beneficial in preventing progression to severe HLH. In Table 2, we compare our case to other reported cases of babesiosis in the literature using the HLH criteria.

**Table 2:** Reported cases of HLH and babesiosis (*Babesia microti*) with our case

Author, year (Ref.)	Auerbach et al. [25]	Gupta et al. [27] Slovut et al. [26]	Poisnel et al. [29]	Mecchella et al. [28]	Our case
Underlying disease	Cryptosporidium	Renal transplant	None	Amyopathic DM and ILD	None
Medication	None	Prednisone, azathioprine	None	Rituximab/MMF/prednisone	Prednisone
Parasitemia %	7	13	3	<1	4
Cytopenia (as per HLH-2004)	No	Yes	Yes	Yes	Yes
Ferritin, ng/mL	Not reported	Not reported	5953	1665	1316
Fibrinogen, mg/dL	Not reported	Not reported	Not reported	Not reported	98
Bone marrow biopsy	Hemophagocytic histiocytes	Hemophagocytic histiocytes	Hemophagocytic histiocytes	Not done	Dyserythropoiesis
LDH, units/L	485	3510	620	586	476
Haptoglobin mg/dL	Not reported	<3	Undetectable	<10	<3
Splenomegaly	Yes	Asplenic		Not reported	No
Fever	Yes	Yes	Yes, value not reported	Yes, but low grade	Yes
Coombs test	Not reported	Positive	Not reported	Negative	Negative

Hematologic manifestations of Babesia are common in the human host. Thrombocytopenia is one of its major features. It is usually caused by hypersplenism which results in increased platelet sequestration and destruction by splenic macrophages [30]. In severe babesiosis, thrombocytopenia could be secondary to DIC. Immune mediated destruction of platelets has also been reported along with autoimmune hemolytic anemia [31]. Shatzel et al. reported two cases of Evans syndrome, the first one had a history of Hodgkin’s lymphoma in remission, along with a history of autoimmune hemolytic anemia 12 years prior to presentation which was treated with splenectomy; he presented again with severe AIHA and thrombocytopenia, but this time after getting infected with Babesia [31]. The second patient had a history of Evans Syndrome treated with splenectomy, but relapsed three weeks post-splenectomy after a babesial infection [31]. Many other case reports described AIHA in patients with babesiosis; this leads us to conclude that there might be an element of immune deregulation precipitated by Babesia [19, 31–34].

Anemia (defined her as hemoglobin ≤10 g/dL) is another well documented hematologic abnormality in babesiosis. Its presence is associated with further complications [19]. Anemia happens after the egress of the parasite from RBCs causing lysis. Hemolysis alone does not really explain the severity of the anemia, since it is more pronounced than the level of parasitemia usually. Otsuka et al. found that cultures of parasitized RBCs of dogs with *Babesia gibsoni* had a significantly higher production of superoxide, which indicates that lipid peroxidation was greater in the infected cells and thus clarifying the role of oxidative damage in host erythrocytes; this could also be the case in human RBCs [35, 36]. There could also be an element of autoimmune hemolysis triggered by Babesia as described previously. In addition, the reticulocyte count is usually high to compensate for hemolysis, however, it can also be low as in our patient suggesting a bone marrow complication like HLH. If the patient has severe anemia, partial or complete red cell exchange transfusion is recommended as per the guidelines of the IDSA in the context of high levels of parasitemia (≥10%); or if the patient has any signs of end organ damage [13].

White blood cell involvement can also happen. Lymphopenia is a common finding among patients with babesiosis, one report described 17 patients with documented babesiosis; among them 13 where identified as lymphopenic [37]. Neutropenia on the other hand is not listed as a common hematologic finding associated with babesiosis [4]. On this contrary some clinicians may view neutropenia as inconsistent with babesiosis leading them to search for another cause [38]. One report assessed the frequency of neutropenia among 51 adult patients who were diagnosed with babesiosis between 2010 and 2013, 18 of them had neutropenia defined as an absolute neutrophil count ≤1800 neutrophils/µL [38]. Mechanisms of WBCs involvement is yet to be clarified whether it’s a result of direct damage to the hematopoietic precursor cells, increased neutrophil adherence, splenic sequestration or a combination of all. In fact a high WBC count i.e. more than 5 × 10^9^/L is a strong predictor of severe babesiosis [11].

The spleen is a vital organ in clearing erythrocytes infected with Babesia, and its absence is a major risk factor for severe infection. The role of the spleen can be illustrated by the mechanism of sequestration, since the parasitized erythrocytes lack the deformability needed to transit the splenic sinusoids and are therefore sequestered within the spleen by resident macrophages [39]. Complications involving this organ can happen, more specifically splenic infarction or rupture. In reviewing the literature we found 11 cases with this complication. Table 3 summarizes these cases.

**Table 3:** Reported cases of splenic rupture due to babesiosis

Author, year (Ref.)	Splenomegaly	Immunity status	Urgent splenectomy
Siderits et al. [39]	Had splenomegaly	Immunocompetent	Yes
Florescu et al. [40]	2 cases both had splenomegaly	2 cases both are immunocompetent	No
Kuwayama and Briones [41]	Subclinical splenomegaly	Immunocompetent	Yes
Froberg et al. [42]	Had splenomegaly	Immunocompetent	Yes
Reis et al. [43]	Not reported	Immunocompetent	Treated by splenic artery embolization
El Khoury et al. [44]	2 cases both had splenomegaly	2 cases both are immunocompetent	No
Tobler Jr. et al. [45]	Had splenomegaly	Immunocompetent	No
Seible et al. [46]	Not reported	Immunocompetent	No
Farber et al. [47]	Subclinical splenomegaly	Immunocompetent	Yes

Accordingly, we conclude that babesiosis can cause splenic rupture, just like malaria which is a well documented cause of this pathology. Mechanisms for malarial splenic rupture may also apply to babesiosis. During infection, pro-inflammatory cytokines are released, specifically TNF, IL-1, IL-6 and IFN-g leading to increased expression of adhesion molecules on the surface of the vascular endothelium. This in turn results in cytoadherence of the infected erythrocytes to the vascular endothelium causing erythrocyte sequestration and obstruction of the vascular flow [48]. In addition, hyperplasia of the reticuloendothelial system may lead to sub-capsular hemorrhage, and eventual splenic capsule breakdown with rupture into the peritoneoum [46]. Conversely, one report examined tissue sections from a splenectomized patient who died from multi-organ failure resulting from severe babesiosis. None of the parasitized erythrocytes examined were close enough to the vascular walls to suggest any degree of sequesteration [49]. Animal models confirmed sequestration of infected erythrocytes (with *B. gibsoni* and Babesia *WA*-*1*) over the capillary endothelial cells [50, 51]. This has to be clarified by more histological sections in the human host. It is reasonable to conclude from published cases of splenic rupture that this is a complication of the immunocompetent individual. Since all cases had some degree of splenomegaly, it is possible to hypothesize that the development of subclinical splenomegaly due to splenic erythrophagocytosis may render the spleen more susceptible to spontaneous rupture with minor trauma [39, 42].

## Conclusion

The incidence of human babesiosis is increasing in the United States, mainly in endemic regions like the Northeastern part. The present case illustrates the importance of considering blood-borne infections in patients presenting with febrile hemolytic anemia. We conclude that human babesiosis is a well documented cause of pancytopenia, hemolysis, splenic rupture and should be considered as a potentially treatable cause of HLH.

## Abbreviations

AIHA: autoimmune hemolytic anemia
ALC: absolute lymphocyte count
ANC: absolute neutrophile count
CBC: complete blood count
CMV: cytomegalovirus
DIC: disseminated intravascular coagulopathy
EBV: epstein barr virus
Hb: hemoglobin
HIV: human immunodefieicny virus
HLH: hemophagocytic lymphohistiocytosis
IDSA: Infectious Diseases Society of America
IFA: immunofluorescence assay
IL: interleukin
ILD: interstitial lung disease
LDH: lactate dehydrogenase
MCV: mean corpuscular volume
NK: natural Killer
PCR: polymerase chain reaction
RBC: red blood cells
WBC: white blood cells
